# Integrative medicine using East Asian herbal medicine for inflammatory pain in patients with rheumatoid arthritis: A protocol for systematic review and meta-analysis integrated with multiple data mining for core candidate discovery

**DOI:** 10.1097/MD.0000000000033903

**Published:** 2023-06-09

**Authors:** Hee-Geun Jo, Jihye Seo, Eunhye Baek, Ji-Hye Hwang, Donghun Lee

**Affiliations:** a Department of Herbal Pharmacology, College of Korean Medicine, Gachon University, Seongnam, Republic of Korea; b Naturalis Inc., Seongnam, Republic of Korea; c Allbarun Kyunghee Korean Medicine Clinic, Gimpo, Republic of Korea; d RexSoft Inc., Seoul, Republic of Korea; e Department of Acupuncture and Moxibustion Medicine, College of Korean Medicine, Gachon University, Seongnam, Republic of Korea.

**Keywords:** bioinformatics, herbal medicine, integrative medicine, meta-analysis, rheumatoid arthritis, systematic review

## Abstract

Rheumatoid arthritis is a chronic inflammatory autoimmune disease characterized by a wide range of clinical symptoms affecting various bodily functions, including skeletal, vascular, metabolic, and cognitive functions. This review aimed to evaluate the efficacy and safety of integrative medicine (East Asian herbal medicine combined with conventional medicine) used for the treatment of inflammatory pain in rheumatoid arthritis and to identify key candidate drugs based on the data.

**Methods:**

A comprehensive literature search will be conducted in 4 core databases (PubMed, Excerpta Medica database, Cochrane Library, and Cumulative Index to Nursing & Allied Health Literature) 4 Korean databases (Oriental Medicine Advanced Searching Integrated System, Korean Studies Information Service System, Research Information Service System, and Korea Citation Index), 2 Chinese databases (Chinese National Knowledge Infrastructure Database and Wanfang data), and 1 Japanese database (Citation Information by National Institute of Informatics) for randomized controlled trials from December 13, 2022. Statistical analysis will be performed using R version 4.1.2 and R Studio program. The American College of Rheumatology 20/50/70 score and rate of adverse events will be the primary outcomes. All outcomes will be analyzed using a random-effects model to produce more statistically conservative results. Sensitivity, meta-regression, and subgroup analyses will be used to identify the sources of any heterogeneity in the study. The revised tool for assessing the risk of bias in randomized trials, version 2.0, will be used to evaluate methodological quality. The overall quality of evidence will be assessed according to the Grading of Recommendations Assessment, Development, and Evaluation Pro Framework.

**Ethics and dissemination:**

There are no ethical issues, as no primary data will be collected directly from the participants. The results of this review will be reported in a peer-reviewed scientific journal.

**Trial registration:**

PROSPERO registration number: CRD42023412385.

**Strengths and limitations of this study**

• Only randomized controlled clinical trials on the use of East Asian herbal medicine as integrative medicine, not monotherapy, will be considered in this study.
• To provide more robust evidence, sensitivity analyses, meta-regressions, and subgroup analyses are used.
• Among other rheumatoid arthritis symptoms, the effect of integrative medicine using East Asian herbal medicine on inflammatory pain in rheumatoid arthritis patients will be explored.
• Based on bioinformatics analysis of the collected drug data, further pharmacological discoveries and mechanisms of action are developed to support therapeutic effects.

## 1. Introduction

Rheumatoid arthritis (RA) is a chronic inflammatory autoimmune disease characterized by a wide range of systemic clinical symptoms that affect skeletal, vascular, metabolic, and cognitive functions.^[1,2]^ In a recent epidemiological study, the annual incidence rate of RA in 2017 was reported to be 14.9%, indicating an 8.2% increase from 1990; moreover, the prevalence and incidence of RA are on the increase worldwide.^[3,4]^ RA is characterized by clinical symptoms, such as pain, swelling, and irreversible joint destruction caused by the infiltration of T and B cells into the synovial membrane of multiple joints and resulting in inflammation.^[5,6]^ RA is associated with several systemic comorbidities.^[7]^ Compared to the general population, patients with RA have a 50% increase in mortality due to cardiovascular morbidity and a decreased life expectancy; therefore, this disease should be considered a serious public health problem.^[4,8]^ These systemic findings and the increased mortality of RA are attributed to long-term progressive inflammation that has a chronic course triggered by autoimmune abnormalities.^[5]^ Moreover, inflammation itself is responsible for up to a fourth of mortalities in patients with RA.^[9]^ Therefore, treatment strategies for RA need to consider the complex pathophysiology unique to immune-mediated inflammatory diseases in a multifaceted manner.

Currently, disease-modifying antirheumatic drugs (DMARDs) are the first-line agents that improve the overall pathology of RA and prevent deterioration.^[10]^ In addition, nonsteroidal antiinflammatory drugs and glucocorticoids are used as symptomatic therapies to suppress symptoms, such as pain.^[2,11,12]^ DMARDs can be classified into synthetic and biological agents. As conventional synthetic DMARDs, methotrexate (MTX), leflunomide, sulfasalazine, and hydroxychloroquine are the most widely used drugs.^[13]^ These drugs are difficult to replace in the treatment of RA; however, various safety issues have not been completely resolved.^[14]^ Even MTX, which has the best efficacy and safety, has been reported to have various adverse effects, including hepatotoxicity, gastrointestinal toxicity, teratogenicity, and pneumonitis due to its cytotoxic properties.^[15,16]^ Furthermore, DMARDs other than MTX may aggravate vascular complications of RA or exhibit harmful cardiovascular effects.^[17]^ In contrast, symptomatic drugs, such as DMARDs and glucocorticoids, have also been reported to have subsafety concerns, such as increasing the risk of infection even at low doses.^[18]^ Moreover, patients who are unresponsive despite receiving active therapies, such as multiple DMARD combination therapy, are also a significant challenge in RA treatment. In this regard, the European Alliance of Associations for Rheumatology also defined such cases as “difficult to treat” RA in 2020. Furthermore, the prevalence of refractory RA among patients has been reported to be up to 10–20% depending on the study.^[19–21]^ In this context, the remaining unmet medical needs in RA treatment at this point can be summarized as improving safety in long-term treatment and preparing a treatment strategy for “difficult to treat” RA.^[22]^

Natural products and phytochemicals are considered promising RA treatment interventions owing to their extensive antiinflammatory and immunomodulatory activities via unique multicompound/multitarget mechanisms.^[23–25]^ In particular, East Asian herbal medicine (EAHM) is currently the most actively studied. In East Asian countries, such as China, Korea, Taiwan, and Japan, more than 500 common EAHM natural products are listed in each country’s pharmacopeia and are still actively used as treatment agents for various acute and chronic diseases.^[26–30]^ Narrowing the scope to RA, the authors investigated whether even when EAHM is used as a monotherapy, it can lead to improvement in inflammatory pain in RA based on its effects on multiple mechanisms and action pathways.^[31]^

Integrative medicine (IM) through combination therapy with conventional intervention is a more typical approach of using EAHM for chronic intractable disorders, such as RA.^[32–35]^ Recently, a number of statistical evidence and real world data have been accumulated to support the potential usefulness of IM for inflammatory and immunological improvement in patients with RA.^[21,36–40]^ For example, in a population-based cohort study in 2020, it was observed that the application of IM could lower the risk of coronary artery disease in RA. Additionally, in a more recent meta-analysis, it was reported that IM could attenuate the adverse effects of DMARDs.^[36,39]^ Considering these circumstances, IM is expected to be worth investigating as a new treatment strategy that can lead to improved treatment results for RA.

However, there are 2 difficulties to be solved when applying IM to RA. First, despite the rapid increase in IM-related research data over the past 10 years, there is still a lack of studies that have reached firm conclusions and reflect the findings of other relevant studies. Second, IM using EAHM is administered under the principle of personalized administration in a wide variety of forms; therefore, it is difficult to reach a consensus on the most useful administration strategy. Based on their understanding of previous studies, the authors aimed to conduct a systematic review of randomized controlled clinical trials (RCTs) with the goal of informing decision-making regarding the potential use of IM using EAHM as a treatment for inflammatory pain in patients with RA. Furthermore, additional multiple data mining of EAHM prescription data gathered through this review will produce hypotheses about potential candidates for the best IM for RA. Through this study, the authors attempted to develop a system for locating valuable candidate materials based on a thorough review of trustworthy data.

## 2. Methods

### 2.1. Study registration

The protocol of this systematic review was registered in in PROSPERO (registration number: CRD42023412385 Available from: https://www.crd.york.ac.uk/prospero/display_record.php?ID=CRD42023412385). This protocol was in accordance with the Preferred Reporting Items for Systematic Review and Meta-Analysis (PRISMA) Protocols 2015, and a systematic review will be conducted following the PRISMA 2020 statement.^[41,42]^

### 2.2. Search strategy

To search for related RCTs, we will conduct a search of the following 11 electronic databases from their inception until December 13, 2022: 4 core databases (PubMed, Excerpta Medica database, Cochrane Library, and Cumulative Index to Nursing & Allied Health Literature), 4 Korean databases (Oriental Medicine Advanced Searching Integrated System, Korean Studies Information Service System, Research Information Service System, and Korea Citation Index), 2 Chinese databases (Chinese National Knowledge Infrastructure Database and Wanfang data), and 1 Japanese database (Citation Information by National Institute of Informatics). The following keywords will be used for the search: rheumatoid arthritis, randomized controlled trial, herbal medicine, traditional Korean medicine, traditional Chinese medicine, and traditional oriental medicine. The complete search strategy for PubMed is ((rheumatoid arthritis[mesh] OR ((rheumatoid OR reumatoid OR rheumatic OR reumatic OR rheumat* OR reumat*) AND (arthrit* OR artrit* OR diseas* OR condition* OR nodule*))[tw] OR (felty* syndrome)[tw] OR (caplan* syndrome)[tw] OR (sjogren* syndrome)[tw] OR (sicca syndrome)[tw] OR “still* disease”[tw] OR “bechterew* disease”[tw]))) AND ((randomized controlled trial[pt] OR controlled clinical trial[pt] OR random*[tiab] OR placebo[tiab] OR clinical trials as topic[mesh] OR trial*[ti]) NOT (animals[mesh] NOT humans[mesh])) AND (“Plants, Medicinal”[mesh] OR “Drugs, Chinese Herbal”[mesh] OR “Medicine, Chinese Traditional”[mesh] OR “Medicine, Kampo”[mesh] OR “Medicine, Korean Traditional”[mesh] OR “Herbal Medicine”[mesh] OR “Prescription Drugs”[mesh] OR “traditional Korean medicine”[tiab] OR “traditional Chinese medicine”[tiab] OR “traditional oriental medicine”[tiab] OR “Kampo medicine”[tiab] OR herb*[tiab] OR decoction*[tiab] OR botanic*[tiab]).

### 2.3. Study selection and Eligibility criteria

**Figure 1.**
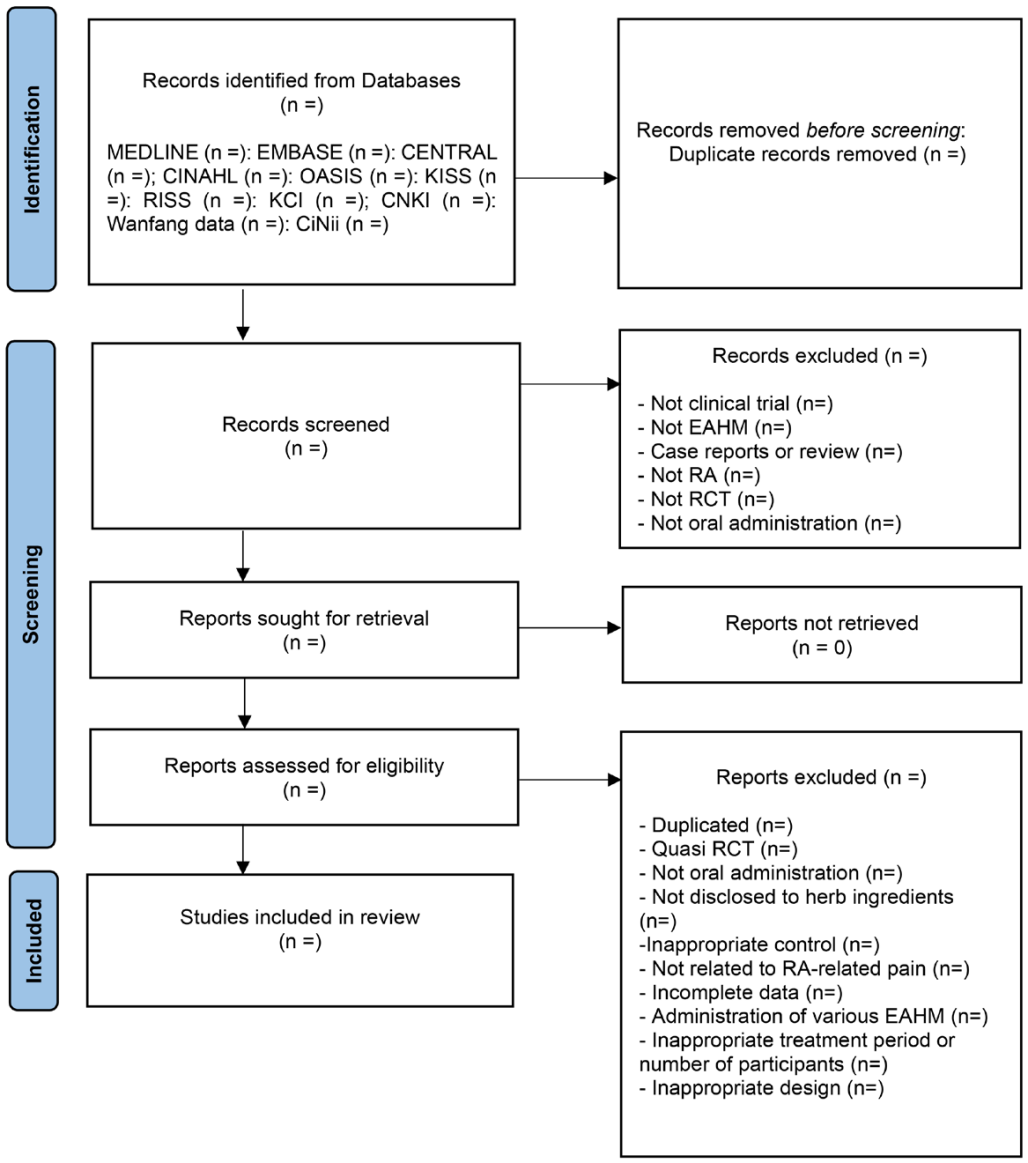
Preferred Reporting Items for Systematic Reviews and Meta-Analysis 2020 flow diagram.

The full texts of the selected studies will be assessed for final inclusion after screening the searched studies using their titles and abstracts. The research selection procedure will be performed independently by 2 authors, using the eligibility criteria listed below. A discussion with other researchers will be used to resolve disagreements in any of the choices. Employing a PRISMA 2020 flow chart, the selection procedure and grounds for exclusion will be demonstrated (Fig. 1).

### 2.4. Types of studies

Studies included in this review will only be RCTs, without restrictions on publication year or language. Nonclinical studies, quasi-RCTs, case reports, reviews, and unpublished studies in peer-reviewed scientific journals will be excluded.

### 2.5. Types of participants

RA participants, who were diagnosed with RA, will be included, with no restrictions on sex or age. We only accept cases where a formal, expert-consensus RA diagnosis is listed as an inclusion criterion in the study.

### 2.6. Types of interventions

RCTs using IM (EAHM through combination therapy with conventional medicine) as experimental interventions, compared with control interventions of the same conventional medicine as experimental interventions, will be included. Studies that meet the following criteria will be excluded:

1. Studies with a treatment period of less than 4 weeks and with less than 30 patients in each group
2. Studies that used combined interventions with other East Asian medical interventions, such as acupuncture, massage, or nondrug therapy only in the experimental group
3. Studies for which the exact constituent herbs of the EAHM formulation used in the study were not identified
4. Studies involving nonoral EAHM, such as topical medicine or injection

### 2.7. Types of outcomes

The American College of Rheumatology 20/50/70 and rate of adverse events (AEs) will be the primary outcomes in this review.

Secondary outcomes will be as follows:

1. Continuous outcomes of RA pain intensity measured using instruments, such as the visual analogue scale, numerical pain rating scale, joint pain index, and joint pain scale of the Western Ontario and McMaster Universities Osteoarthritis Index.
2. The remission rate of RA pain and inflammation-related symptoms observed according to explicit criteria
3. Series of index groups to evaluate inflammatory findings, such as the tender joint count, swollen joint count, erythrocyte sedimentation rate, and C-reactive protein
4. An index that can evaluate the overall related symptoms that may accompany inflammation and pain in RA and the patient’s physical condition measured using disease activity score 28 and a health assessment questionnaire

### 2.8. Data collection and analysis

#### 1.2.8. Data extraction.

Data from the included studies will be collected into predesigned data extraction forms by 2 independent reviewers. The information extracted from the included studies will be the title, authors’ names, country where the study was conducted, publication year, sample size, participant characteristics (age and sex distribution), interventions, treatment duration, results of outcome index, AEs, and EAHM information. Any discrepancies in this process will be resolved through discussions with other researchers. By default, all outcome data will be summarized and presented in a table. If any of the outcomes are not suitable for quantitative synthesis, they will be described qualitatively in the body of the results based on the table summary.

#### 2.2.8. Risk of bias assessment.

To evaluate the methodological quality of each included RCTs, the Cochrane Collaboration’s modified tool for risk of bias in randomized trials, Rob 2.0, will be used.^[43]^ Two independent reviewers will assess the methodological quality of all included studies, and any disagreements will be resolved by discussion with other researchers. Rob 2.0 includes 5 domains: bias arising from the randomization process, bias deviating from the intended intervention, bias due to omission of outcome data, and bias in the selection of reported outcomes. The quality of each domain will be assigned 3 levels: high risk of bias, low risk of bias, and some concerns.

#### 3.2.8. Data synthesis.

Software R (version 4.1.2, R Foundation for Statistical Computing, Vienna, Austria) and R studio program (version 1.4.1106, Integrated Development for R. R Studio, PBC, Boston, MA) will be used for data synthesis if two or more studies report the same outcome. Using the random-effects model, the effect size and 95% confidence interval (CI) of data from the included RCTs will be calculated. We will calculate heterogeneities using *I*^2^ and χ^2^ test statistics. Heterogeneity was interpreted as statistically significant when *I*^2^ is ≥50% or the *P* value based on χ^2^ test is <.10.

American College of Rheumatology 20/50/70 and remission rate will be analyzed using the relative risk with 95% CIs. AEs will be analyzed using odds ratios. For continuous outcome measures, such as tender joint count, swollen joint count, erythrocyte sedimentation rate, and C-reactive protein, we will calculate mean differences (MD) and their 95% CIs. If there are several types of indicators for the same measurement target, such as RA pain intensity, we will calculate a standardized MD instead of the MD. If heterogeneity was assessed as statistically significant in the synthesized results for the primary outcome, we will perform a subgroup analysis by determining the scope of additional analysis for the following domains: diagnostic criteria, type of comparator, type of EAHM preparation, duration of treatment, and source of investigational medication.

If heterogeneity was detected in the main outcomes of the meta-analysis, further analyses will be performed to confirm the explanation. First, we will perform a sensitivity analysis using a leave-one-out approach to determine whether the included data are affected by outliers. If no outliers are identified in the sensitivity analysis, meta-regression analysis will be performed on the following 7 items specified through the protocol in advance: control drug, treatment period, source of an investigational drug, formulation type, sample size, overall risk of bias, and randomization method, which causes a significant difference in results. A subgroup analysis will also be performed. To assess publication bias, a contour-enhanced funnel plot will be created and any evidence of visual asymmetry will be observed.^[44]^ We will use the Egger and Begg tests to further explore any asymmetry, to investigate whether there can be other reasons for funnel plot asymmetry than reporting bias.^[45,46]^

### 2.9. Quality of evidence assessment plan

The quality of the evidence derived from data synthesis will be assessed according to the Grading of Recommendations Assessment, Development, and Evaluation (GRADE) Pro.^[47]^

### 2.10. Additional analysis of EAHM information

Additional analyses will be conducted to explore key EAHM candidates for IM treatment and reveal mechanistic information supporting their clinical effects.

Through multidata mining analysis of IM medication data, materials that are predicted to have a key pharmacological action will be derived and classified as core candidate EAHM. Afterwards the dosage and duration of the administration range of the core candidate will be investigated separately. The techniques to be used for data mining are as follows:

1. Using a descriptive statistical approach, we will first identify EAHMs that are frequently prescribed in more than 10% of the IM trials included in the review.
2. Through social network analysis,^[48]^ we will select the EAHM in each IM regimen that plays a central role in the relationship between different drugs. This analysis will be done based on the eigenvector centrality value, using the following formula:

$$\mathrm{Ci}=\;\frac{1}{\lambda }\sum_{j\in N\left(i\right)}A_{ij}C_{j}$$

where *N*(*i*) represents the collection of herbs that are close to material *i* and λ is the eigenvalue of material *i*, a constant determined by the algorithm. If materials *i* and *j* are connected in the *n* × *n*-direction adjacency matrix *A, A*_*ij*_ becomes “1”; otherwise, it becomes “0.” Herb *i* and its neighbors constitute herb *j*, which is the eigenvector centrality value of *C*_*j*_. Centrality measurements will be performed on materials that show a frequency of use in more than 5% of the included trials.

1. Association rule mining^[49]^ will be performed to predict combination patterns expected to have pharmacological synergistic effects among all EAHM materials included in the IM prescription. The formula of the evaluation measure used in this analysis method is as follows:

$$\begin{array}{c} support\;\left(A\right)=P\left(A\right)\\ confidence\left(A\to B\right)=\;\text{​}\text{​}\;\text{​}\text{​}\;\frac{p\left(A\cap B\right)}{p\left(A\right)}\\ Lift\left(A\to B\right)=\frac{p\left(A\cap B\right)}{p\left(A\right)\text{*}p\left(B\right)} \end{array}$$

1. Based on the above 3 analyses, any EAHM that is predicted to simultaneously satisfy the 3 conditions of “frequently used in clinical practice,” “central position in individual prescriptions,” and “strong association with other drugs” will be selected as a core candidate EAHM.
2. The selected core candidates will be classified into 2 groups, “the hot properties” and “the cold properties” groups, according to the EAHM indication principle and expected effect. Based on the pharmacological approach, additional information on mechanisms supporting efficacy, major active ingredients, targets, pathways, and toxicity will be derived for each group, presented by the group, and this information will be compared with each other.

### 2.11. Amendments

The details and dates of any revisions will be noted in the final report if the protocol is substantially changed or modified.

### 2.12. Ethics and dissemination

No personally identifiable information or other sensitive data will be published or disclosed as part of this systematic review. The rights of participants will not be violated in this study. Ethical approval is not required as this is not a clinical trial where participants have been directly recruited. The results of this study will be published in a peer-reviewed scientific publication.

## 3. Discussion

This study will be carried out to create translational medical information ranging from efficacy and safety, as well as core materials and action mechanisms, after systematically collecting the maximum range of RCTs that correspond to the efficacy of IM for RA.

Based on this, it is expected that solutions to the following problems can be presented.

First, several studies have demonstrated that IM is a more effective alternative to monotherapy for various diseases including RA.^[21,40]^ However, since only the synthesis of evidence for a relatively limited range has been attempted thus far, there is a lack of confidence in the robustness of the conclusion due to the insufficient sample size. As this study will collect and systematically analyze subject-related studies to the maximum extent, it will be possible to derive more statistically robust decision-making grounds based on a sufficiently large sample size.

Second, EAHM is used as a specially formulated polyherbal formulation, in line with personalized indications.^[30,50]^ As a result, there are inconsistencies in the dosage and composition of IM medications used in the most clinical studies. The heterogeneity observed in a previous meta-analysis was believed to be predominantly caused by this problem. In addition, this characteristic makes it challenging for readers to determine whether a formula in a certain trial is relevant to the optimal IM. Furthermore, the fact that specific information on the optimal prescription was not derived contributes to the problem of making it difficult to produce useful hypotheses for follow-up studies.

Third, EAHM is used in the form of a complex prescription composed of multiple materials and not a single drug.^[31,51,52]^ In addition, even a single material of EAHM exhibits effects based on a complex mechanism of action expressed as a “multicompound-multitarget-multipathway” due to the nature of natural products.^[53,54]^ This is a very different characteristic from single-component synthetic drugs, whose structures and mechanisms of action are already clearly known. Therefore, clinical studies using EAHM to be meaningful, it is necessary to make the most of the available data and precisely analyze information related to the mechanism of action. Fortunately, various machine learning and bioinformatics analysis techniques that have been widely used recently have made it possible to more precisely connect the clinical effect and mechanism of action of EAHM.^[55]^

Through multilateral analysis of the data, it will be possible to predict whether IM using EAHM can become a data pool for the search for high-quality new drug candidates, and which EAHM is more valuable for further research. However, this study is expected to be meaningful in that it provides a translational study by adding improvements considering the characteristics of EAHM to the current clinical research methodology.

## Acknowledgments

We would like to thank Editage (www.editage.co.kr) for English language editing.

## Author contributions

**Conceptualization:** Hee-Geun Jo, Jihye Seo, Eunhye Baek, Ji-Hye Hwang, Donghun Lee.

**Formal analysis:** Hee-Geun Jo, Jihye Seo, Eunhye Baek, Ji-Hye Hwang, Donghun Lee.

**Funding acquisition:** Ji-Hye Hwang, Donghun Lee.

**Investigation:** Hee-Geun Jo, Jihye Seo, Eunhye Baek, Donghun Lee.

**Methodology:** Hee-Geun Jo, Jihye Seo, Eunhye Baek, Ji-Hye Hwang, Donghun Lee.

**Project administration:** Ji-Hye Hwang, Donghun Lee.

**Resources:** Hee-Geun Jo, Jihye Seo, Eunhye Baek, Ji-Hye Hwang, Donghun Lee.

**Software:** Hee-Geun Jo.

**Supervision:** Ji-Hye Hwang, Donghun Lee.

**Validation:** Hee-Geun Jo, Jihye Seo, Eunhye Baek, Ji-Hye Hwang, Donghun Lee.

**Visualization:** Hee-Geun Jo.

**Writing – original draft:** Hee-Geun Jo, Jihye Seo.

**Writing – review & editing:** Hee-Geun Jo, Jihye Seo, Eunhye Baek, Ji-Hye Hwang, Donghun Lee.
